# Increased TNN in Knee Osteoarthritis Accelerates Cartilage Damage via the Negative Regulation of AMPK‐PPARγ Signalling

**DOI:** 10.1111/jcmm.70981

**Published:** 2025-12-14

**Authors:** Zhiyu Chen, Gean Wu, Yizhe Fan, Chao Li, ChenHao Wang, Chengyi Yang, Shixiang Wu, Zhaoshun Wu, Peng Wang, Yafeng Zhang, Wulin You

**Affiliations:** ^1^ Wuxi Affiliated Hospital of Nanjing University of Chinese Medicine Wuxi China; ^2^ Longhua Hospital Shanghai University of Traditional Chinese Medicine Shanghai China

**Keywords:** AMPK, cartilage damage, knee osteoarthritis, PPAR‐γ, tenascin‐W

## Abstract

In this study, we aimed to get a better understanding of which genes are involved in cartilage damage in KOA and the pathological mechanisms. RNA‐seq for cartilage tissues obtained from Normal rats and KOA rats, for IL‐1β‐stimulated chondrocytes and IL‐1β‐stimulated chondrocytes treated with TNN recombinant protein, was conducted respectively. The degree of cartilage injury was evaluated by HE and Safranin O‐fast green staining, and the expression abundance of TNN and p‐AMPK in cartilage tissue or chondrocytes was observed by immunofluorescence. TNN was inhibited in vivo and in vitro by lentivirus and siRNA, respectively. The gene and protein levels of protease MMP3, MMP13, ADAMTS4, and ADAMTS5 and AMPK/PPAR‐γ pathway‐related factors ‐AMPK, AMPK, PPAR‐γ, PGC‐1α, mTOR were detected by PCR and WB, respectively. The mitochondrial membrane potential of chondrocytes was evaluated by JC1 probe, and the oxygen level of chondrocytes was evaluated by ROS immunofluorescence. RNA‐seq revealed TNN was significantly up‐regulated in the KOA group, and DEGs were mainly enriched in ‘extracellular matrix’. Subsequently, TNN inhibition could reduce the expression of MMPs, ADAMTSs were demonstrated in vivo and in vitro. Further on, RNA‐seq on IL‐1β‐stimulated chondrocytes and chondrocytes treated with TNN recombinant protein after IL‐1β stimulation confirmed that AMPK‐PPARγ signalling might be the downstream pathway of TNN, and the negative regulation of TNN on AMPK‐PPARγ signalling was observed in vivo and in vitro. This study innovatively unveils the increased TNN in KOA accelerates cartilage damage, and this damage‐promoting effect is achieved by negative regulation of AMPK‐PPARγ signalling.

**Abbreviations**
ADAMs: a disintegrin and metalloproteinase
ADAMTSs: ADAMs with thrombospondin motifs
AMPK: adenosine 5′‐monophosphate activated protein kinase
ECM: extracellular matrix
IL‐1β: interleukin‐1β
KOA: knee osteoarthritis
MMP: matrix metalloproteinase
mTOR: mammalian target of rapamycin
PGC‐1α: PPAR‐γ coactivator‐1α
PPAR‐γ: peroxisome proliferative activated receptor‐γ
TNF‐α: tumour necrosis factor‐α

## 1 Introduction

Knee osteoarthritis (KOA) is the most common form of arthritis and a major cause of disability and chronic pain, characterised by gradual loss of articular cartilage, synovial membrane inflammation, osteophyte formation and subchondral bone sclerosis [1, 2]. Traditionally, KOA has been considered a disease of cartilage wear and tear; magnetic resonance imaging acquisitions for assessing cartilage thickness are commonly used in clinical practice to assess the severity of KOA [3, 4], emphasising the key role of cartilage damage in the development of KOA. In general, cartilage loss in KOA is caused by multifactorial parameters, including excessive production of matrix degrading enzymes such as matrix metalloproteinases (MMPs) and a disintegrin and metalloproteinase (ADAMs) with thrombospondin motifs (ADAMTSs). They disrupted the benign balance of synthesis and degradation of extracellular matrix (ECM) [5]. These conditions accelerated chondrocyte hypertrophy and increased focal calcification of joint cartilage [6]. Eventually, cells undergo apoptosis, which leads to the destruction of cartilage tissues. However, the molecular mechanisms regulating these processes in chondrocytes remain unclear.

Histologically, chondrocytes are embedded within the matrix and distance themselves from each other by the matrix, thus creating more opportunities for chondrocytes and the matrix to communicate [7]. Chondrocytes regulate cartilage homeostasis partly by synthesising an ECM rich in type II collagen (Col II), proteoglycans and related macromolecules, while the pathological influence of KOA on cartilage ECM may also initiate molecular signal transduction of chondrocytes through biomechanical, chemical and other factors, and affect their biological status [8, 9]. For example, tenascins are a family of large oligomeric ECM glycoproteins, comprising the four family members of tenascin‐C, tenascin‐N (TNN), tenascin‐X and tenascin‐R, with tenascin‐C being the most thoroughly studied in the KOA field [10, 11]. Swahn H, et al. revealed a chondrocyte population expanded in KOA, which has critical roles in ECM and tenascin signalling and is the dominant sender of signals to all other cartilage and meniscus clusters [12]. Previous studies have shown that stimulation of inflammatory factors, including tumour necrosis factor‐α (TNF‐α) and interleukin‐1β (IL‐1β), can up‐regulate TNC and then promote chondrocyte apoptosis and accelerate cartilage degradation of KOA through integrin alpha 9, transforming growth factor β, and other signalling pathways [12, 13, 14]. Besides, the loss of tenascin‐XB in mouse cartilage inhibited phosphorylation of protein kinase B and promoted chondrocyte apoptosis, augmenting cartilage degeneration and subchondral bone loss [15].

In clinic, tenascin‐C levels in the synovial fluid were shown to be significantly increased in KOA patients and correlated with the radiographic grading levels of KOA [16, 17]. Immunohistochemical analysis of tenascin‐C expression revealed that tenascin‐C staining intensity increased with the degeneration of cartilage [18]. Therefore, tenascin‐C has been demonstrated to be a useful marker of KOA. However, we still lack understanding of the specific mechanisms of the tenascins family participating in KOA cartilage damage, especially subtypes other than the tenascin‐C protein. In addition, due to the KOA treatment still lacking possible disease‐modifying osteoarthritis drugs currently, revealing pharmacological targets of these useful markers for disease progression is expected to provide new directions for the treatment of KOA.

In this study, we first employed RNA‐sequencing (RNA‐seq) to analyse differentially expressed genes (DEGs) in cartilage tissues of normal and KOA rats. We identified *TNN*, the gene encoding tenascin‐N protein (also known as tenascin‐W) [19], which was significantly differentially up‐regulated in the KOA group. Subsequently, we confirmed the chondroprotective effect of inhibition of TNN on KOA in vivo and in vitro. In order to further study the downstream target tenascin‐W, chondrocytes were treated with IL‐1β to mimic the inflammatory environment of KOA in vitro, and RNA‐seq was proceeded to KOA chondrocytes with or without TNN intervention. We found DEGs were mainly enriched in adenosine 5′‐monophosphate activated protein kinase (AMPK) and peroxisome proliferative activated receptor‐γ (PPAR‐γ) signalling pathways. Since PPAR is widely considered to be one of the downstream molecules of AMPK [20, 21], we hypothesised that elevated TNN in KOA cartilage may accelerate the cartilage damage by regulating AMPK/PPAR‐γ. Finally, we used molecular biology methods to verify the above hypothesis in vivo and in vitro.

## 2 Material and Methods

### 2.1 Animals and In Vivo Experiment Design

SD male rats, 180 to 220 g, were purchased from Charles River Laboratories and housed in a specific pathogen‐free, laminar‐flow housing apparatus under 25°C ± 2°C temperature, 55% ± 5% humidity, 12 h light/dark regimen. All animal protocols were approved by the Animal Care and Use Committee of the Wuxi Hospital Affiliated to Nanjing University of Chinese Medicine, No. 2024 (Animals)‐030‐1.

The rat KOA model was constructed by anterior cruciate ligament transection (ACLT) surgery according to previous studies [22], both knees, and 14 days after the surgery, the modelling was established successfully. Cartilage tissue from 3 blank rats and 3 KOA rats was taken for RAN‐seq. In another set, 24 rats were randomly divided into the Normal group, KOA group, KOA + TNN group and KOA + TNN siRNA group (*n* = 6 per group). Except for the Normal group, all the other groups were treated with ACLT. The KOA + TNN group received TNN recombinant protein 5 μg in 50 μL saline intraarticular injection once a week for 2 weeks according to the reagent instructions and the research of Liu L et al. after the KOA model was constructed [23], while the KOA + TNN siRNA group received commercial lentivirus mediated TNN siRNA 20 μL (1 × 10^8^ TU/mL, RiboBio, China) for intraarticular injection once a week for 2 weeks.

### 2.2 Cell Culture and In Vitro Experiment Design

Primary rat chondrocytes were extracted as the method described previously [24]. Briefly, cartilage tissues from the distal femur and the proximal tibia of rats were harvested. The cells were digested with 0.2% collagenase type II at 37°C for 8 h, and the digestion was terminated by the addition of 10% foetal bovine serum (Gibco, USA). Cells were cultured in complete medium made of DMEM/F12 medium supplemented with 10% FBS and 1% penicillin and streptomycin and incubated under standard conditions (37°C, 5% CO_2_), and were used for up to five generations in all vitro experiments.

To simulate the inflammatory environment of KOA in vitro, chondrocytes were treated with IL‐1β 10 ng/mL for 6 h (KOA group). The KOA + TNN group was treated with TNN recombinant protein 10 ng/mL for 24 h after IL‐1β stimulation, while the KOA + TNN siRNA group required transfection with commercial TNN siRNA (RiboBio, China) prior to the intervention of IL‐1β. In brief, chondrocytes were transfected using Lipofectamine 2000 at a concentration of 100 nM according to the manufacturer's protocol. The transfection efficiency was determined using qPCR and Western blotting after being transfected for 24 h.

### 2.3 RNA‐Seq

RNA‐seq was performed on the cartilage tissues of the Normal group and KOA group, and on the chondrocytes of the KOA group and KOA + TNN group, respectively. Total RNA was extracted using the EZ‐press RNA Purification Kit according to the manufacturer's protocol, and RNA purity and quantification were evaluated using the Nano Drop 2000 spectrophotometer (Thermo Scientific, USA). RNA integrity was assessed using the Agilent 2100 Bioanalyzer (Agilent Technologies, USA). The samples with an RNA Integrity Number of 7 were subjected to the subsequent analysis. Then, the libraries were constructed using the TruSeq Stranded mRNA LT Sample Prep Kit (Illumina, USA) according to the manufacturer's instructions. These libraries were sequenced on the Illumina sequencing platform (HiSeq TM3000), and 125 bp/150 bp paired‐end reads were generated.

The resulting reads were aligned, quantified, filtered and analysed for differential expression. For each contrast extracted with Limma, global perturbations in known GO enrichment and KEGG pathways analysis were performed using, respectively, the R/Bioconductor package. Gene set enrichment analysis (GSEA) was performed using GSEA software (Broad Institute, USA). Genes were ranked according to their expression; gene sets were searched from www.gsea‐msigdb.org.

### 2.4 Histological Analysis

Rat knee joints were fixed in 4% PFA and decalcified in 0.5 mol/L EDTA (FUJIFILM Wako pure Chemical, Japan) for 3 weeks. Articular cartilage samples were then embedded in paraffin, sectioned to 5 μm thickness, stained with HE, Safranin O‐fast green, according to the instructions of the stain kit, respectively. Sections were observed under a Leica DMI3000B microscope, using bright field.

### 2.5 Immunofluorescence

Cartilage sections heated at 95°C for 15 min and treated with 3% H_2_O_2_, or chondrocytes fixed in 4% paraformaldehyde at room temperature for 30 min, both of which were brightened with 0.5% Triton X‐100/PBS for 30 min, then blocked with 5% BSA for 1 h at room temperature. Sections were incubated with primary antibodies TNN (Abcam, UK) and p‐AMPK (Abcam, UK) overnight at 4°C, and after extensive washing, incubated with the corresponding fluorescent secondary antibody. The nucleus was stained with DAPI for 10 min at room temperature. Sections were observed using a laser confocal microscope (Zeiss LSM710, Germany).

### 2.6 Determination of the ΔΨm

ΔΨm (mitochondrial membrane potential) was measured using a JC‐1 probe kit (Beyotime, China). JC‐1 would form a polymer (J‐aggregates) that produces red fluorescence (Ex/Em = 585/590 nm) when ΔΨm was high and exists as a monomer (J‐monomer) to produce green fluorescence (Ex/Em = 510/527 nm) when ΔΨm was low. Chondrocytes were incubated with JC‐1 staining solution for 20 min, gently washed with PBS, and resuspended in serum‐free medium. Images were acquired using a Zeiss confocal laser scanning microscope, and further analysis of the fluorescence intensity was performed using Zeiss LSM Image Examiner software. The ΔΨm was represented by the ratio of red to green fluorescence.

### 2.7 Assessment of the Mitochondrial ROS Levels

The total ROS levels in chondrocytes were measured using an ROS Assay Kit (Beyotime, China) following the manufacturer's protocol. Mitochondria were located through MitoBright LT Deep Red (Ex/Em = 650/700 nm, DOJINDO Laboratories, China). Briefly, cells were incubated with 100 nmol/L MitoBright reagent working solution in the dark for 10 min at 37°C. After gentle washing, images were acquired using a Zeiss confocal laser scanning microscope, and further analysis of the fluorescence intensity was performed using Zeiss LSM Image Examiner software.

### 2.8 Enzyme Linked Immunosorbent Assay (ELISA)

The content of MMP3, MMP‐13, and ADAMTS4, ADAMTS5 in serum or in the culture media was determined using a commercially available rat ELISA kit (Beyotime Biotechnology, China) according to the manufacturer's instructions.

### 2.9 Real‐Time PCR

Total RNA from chondrocytes was extracted by using TRIzol reagent (Invitrogen, USA) according to the manufacturer's instructions, and was synthesised to cDNA by reverse transcription (cDNA synthesis kit, Takara, Japan). Power SYBR Green PCR Master Mix (Applied Biosystems, USA) was used for real‐time PCR. The mRNA levels of MMP3, MMP13, ADAMTS4, ADAMTS5, mTOR, PPAR‐γ and PGC‐1α were analysed using primer sequences listed in Table 1. Real‐time PCR was performed using Premix Ex Taq SYBR‐Green PCR (Takara Biotechnology, Japan) according to the manufacturer's instructions on an ABI PRISM 7300 (Applied Biosystems, USA). The mRNA level of individual genes was normalised to GAPDH and calculated by the 2^−ΔΔCT^ data analysis method.

**TABLE 1:** Nucleotide sequences of primers used for real‐time PCR amplification.

Target gene	Forward primer	Reverse primer
MMP3	CAGCCTGAGGCTCTCTCTGT	TGAGGCTCAGGTTCTCTGTT
MMP13	CTGACCTGGGTTGAGGAGAG	CCTTCCACGACATTCAGGAA
ADAMTS4	TGATGCCTGCTGTCACTGTG	AGCCTCCGCTGATGATCTTC
ADAMTS5	TGCGGAGTTGAGTTCTACGA	CCTCTGCTGCTGTACTGGTG
mTOR	AGCCTGAGGACCACCTTTTC	GGTCTGCCAGCCTCTAGTGA
PPAR‐γ	AAGCAGCACTGCTCAACGAG	CCTCCTCCGTCTTCCTCTTG
PGC‐1α	GCGGTTTGCTCACTGTTCTG	CAGGAGGGTGGTAGGTGTTG
GAPDH	ACCACAGTCCATGCCATCAC	TCCACCACCCTGTTGCTGTA

### 2.10 Western Blotting (WB)

Total proteins were obtained by adding RIPA lysate, and the protein concentration was measured in accordance with the instructions for the preparation of the BSA standard curve. Then, according to the determination of protein concentration and the volume of the sample on the sample, electrophoresis, membrane transfer, BSA closed, add the corresponding primary antibody (1:1000), incubated with secondary antibody, and exposed.

### 2.11 Statistical Analysis

All experiments were performed independently at least thrice, and data were presented as mean ± standard deviation. Statistical analysis was performed using GraphPad Prism 8.0 Software. Group comparisons were assessed with Student's *t*‐test or one‐way ANOVA for the comparison of multiple columns. A value of *p* < 0.05 (two‐tailed) was considered statistically significant.

## 3 Results

### 3.1 RNA‐Seq of KOA Rat Cartilage Revealed TNN Was Closely Related to the Change of Extracellular Matrix Environment

**FIGURE 1:**
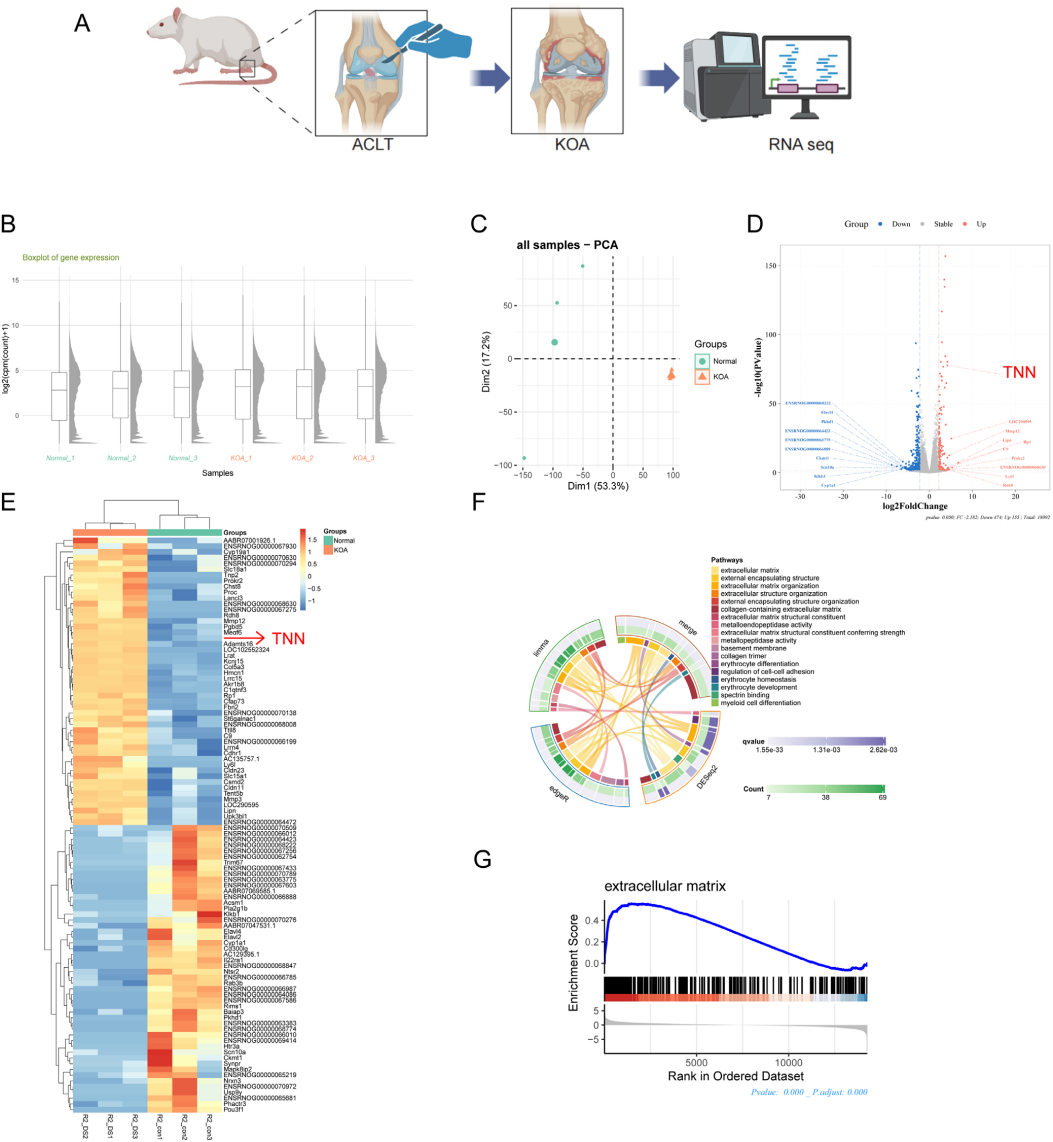
RNA‐seq of KOA rat cartilage revealed TNN was closely related to the change of extracellular matrix environment. (A) Schematic diagram of the experiment. (B) The boxplot diagram reflected the sequencing depth of each tissue sample. (C) Individual‐PCA plot of gene expression in Normal (cyan) and KOA samples (orange) revealed strong clustering of samples by phenotype. (D) Volcano plot representation of DEGs analysis in KOA and Normal. (E) DEGs heat map of KOA and Normal. (F) GO analysis conducted by DEGs of KOA and Normal. (G) GSEA analysis of DEGs in ‘extracellular matrix’.

To better understand which genes are involved in cartilage damage in KOA, we performed RNA seq for cartilage tissues obtained from the Normal group and KOA group (Figure 1A). The boxplot (Figure 1B) reflected the sequencing depth of the samples is consistent. The principal components analysis (PCA, Figure 1C) revealed strong clustering of samples by phenotype. Compared with the Normal, the cartilage tissue of the KOA group identified 155 up‐regulated genes and 474 down‐regulated genes, and TNN was significantly different and marked in the volcano map (Figure 1D). Up‐regulated DEGs between the two groups were also included Cyp19a1, Slc18a1, Tnp2, Prokr2, Mmp12, Adamts16, and so on (Figure 1E), and Go analysis (Figure 1F) showed that DEGs were mainly enriched in ‘extracellular matrix’, ‘extracellular matrix organization’, ‘collagen‐containing extracellular matrix’, ‘extracellular matrix structural constituent’, and other pathways. GSEA plots evaluating the changes of extracellular matrix‐related genes, and genes in the extracellular matrix disassembly category were upregulated (Figure 1G).

### 3.2 Inhibition of TNN Alleviated the Progression of Cartilage Damage in KOA Rats

**FIGURE 2:**
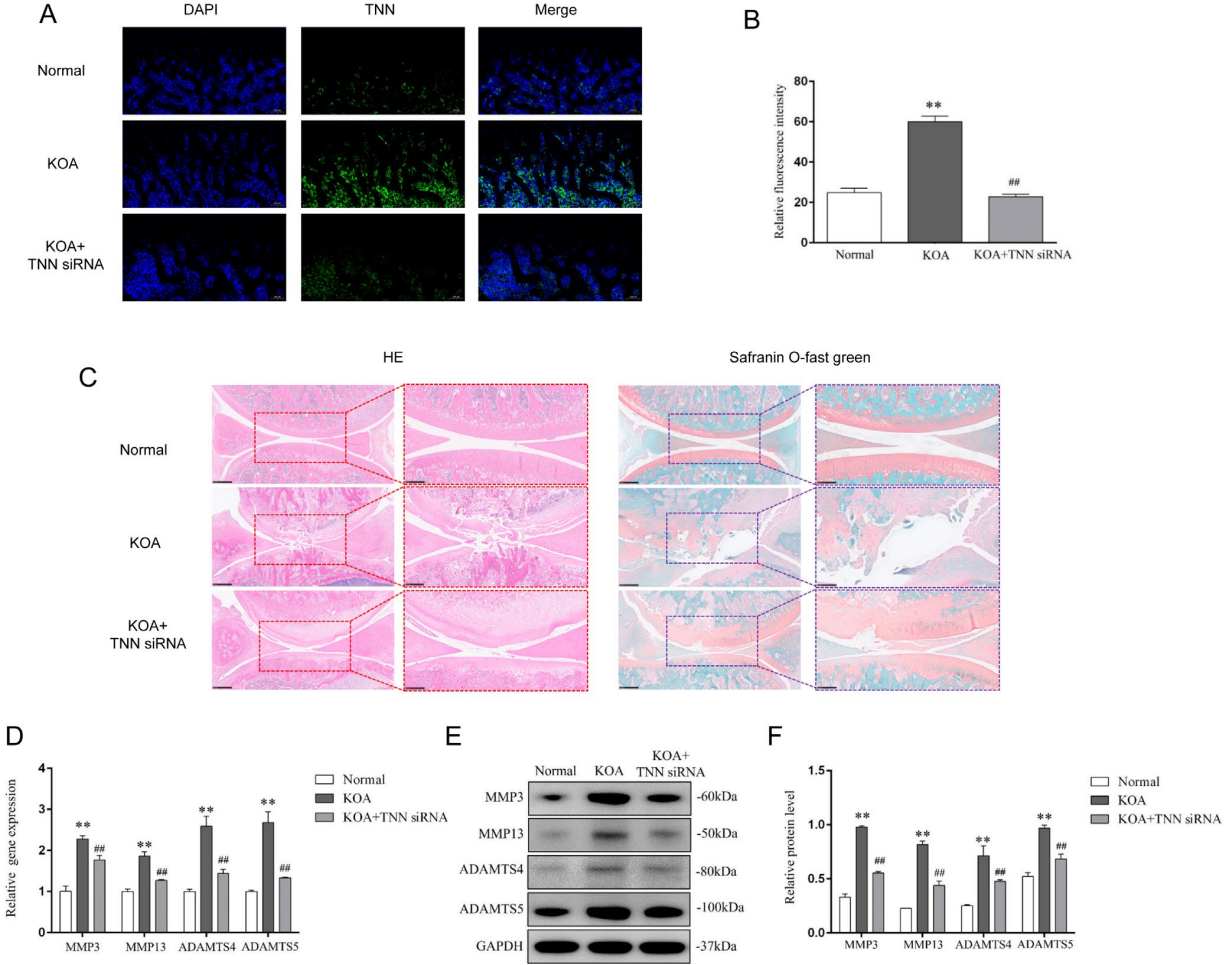
Inhibition of TNN alleviated the progression of cartilage damage in KOA rats. (A) Representative TNN immunofluorescence of cartilage in each group, 100×, scale bar = 200 μm. (B) Relative fluorescence intensity of TNN. **p < 0.01 vs. the Normal groups, ^##^ *p* < 0.01 vs. the KOA. (C) Representative HE and Safranin O‐fast green staining of cartilage tissues in each group, 100×, scale bar = 500 μm. (D) Relative gene expression of MMP3, MMP13, ADAMTS4 and ADAMTS5 in cartilage. **p < 0.01 vs. the Normal groups, ^##^ *p* < 0.01 vs. the KOA group. (E) Typical protein bands of MMP3, MMP13, ADAMTS4 and ADAMTS5. (F) Relative protein level of MMP3, MMP13, ADAMTS4 and ADAMTS5 in cartilage. **p < 0.01 vs. the Normal groups, ^##^ *p* < 0.01 vs. the KOA group.

Next, we investigated whether TNN inhibition can alleviate the cartilage damage of KOA in vivo. Immunofluorescence was performed to label TNN protein in cartilage tissue (Figure 2A,B), and in consistency with the results of RAN‐seq in cartilage, the relative fluorescence intensity of TNN was increased in the KOA group compared with the Normal (*p* < 0.01), and the intervention of lentivirus‐mediated TNN siRNA significantly decreased TNN fluorescence intensity (*p* < 0.01). Besides, the articular cartilage of the KOA group showed more inflammatory cell infiltration, a rough and fractured surface, and more collagen staining lost, but these pathological changes were alleviated in the KOA + TNN siRNA group (Figure 2C). In addition, both gene and protein levels (Figure 2D–F) of MMP3, MMP13, ADAMTS4 and ADAMTS5 were up‐regulated in the KOA group compared with the Normal group (*p* < 0.01), and down‐regulated in the KOA + TNN siRNA group compared with the KOA (*p* < 0.01), suggesting the inhibition of TNN alleviated the progression of cartilage damage in KOA rats.

### 3.3 Silencing TNN Reduced the Release of MMPs and ADAMTSs From KOA Chondrocytes

**FIGURE 3:**
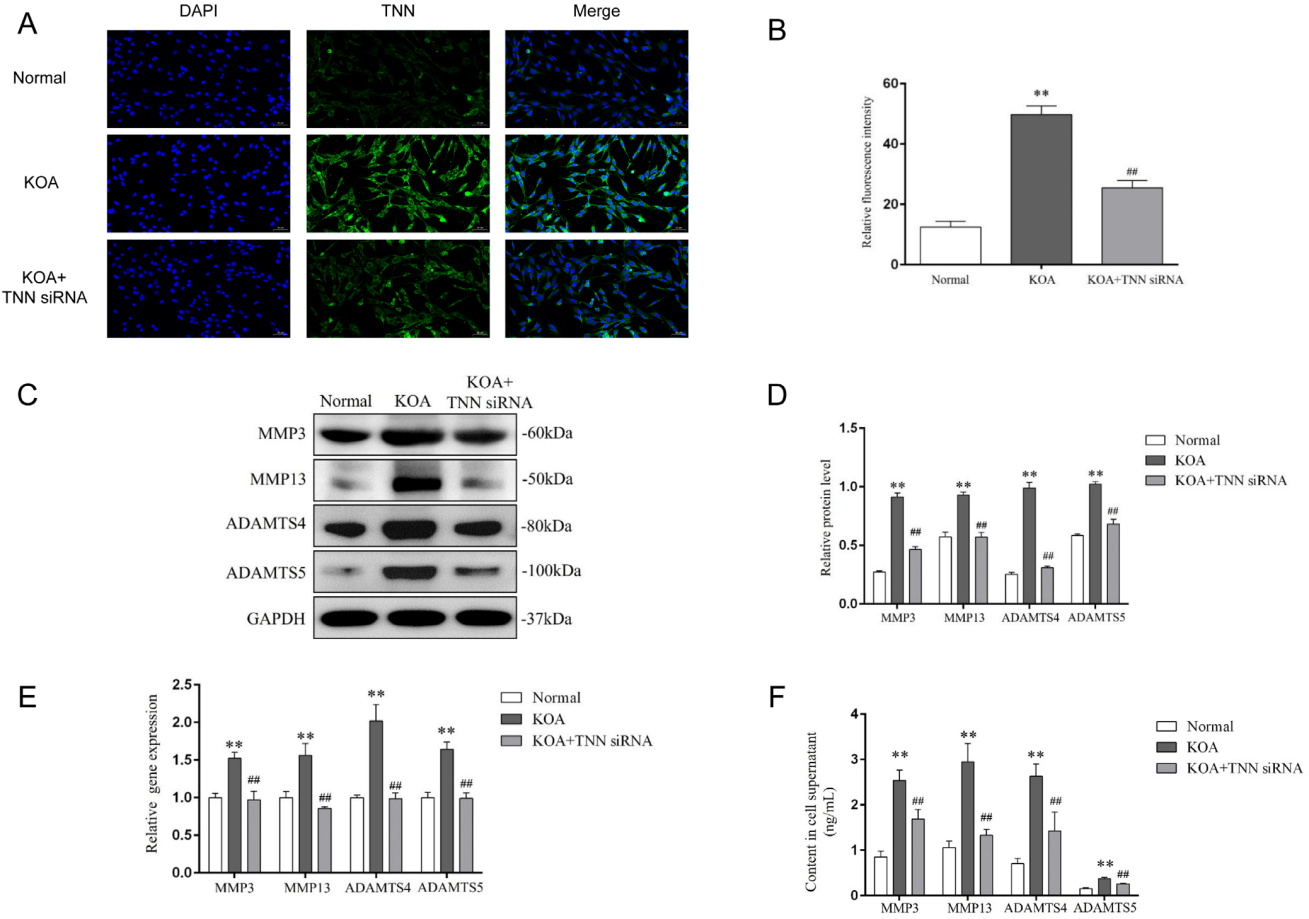
Silencing TNN reduced the release of MMPs and ADAMTSs from KOA chondrocytes. (A) Representative TNN immunofluorescence of chondrocytes in each group, 400×, scale bar = 50 μm. (B) Relative fluorescence intensity of TNN. **p < 0.01 vs. the Normal groups, ^##^ *p* < 0.01 vs. the KOA. (C) Typical protein bands of MMP3, MMP13, ADAMTS4 and ADAMTS5. (D) Relative protein level of MMP3, MMP13, ADAMTS4 and ADAMTS5 in chondrocytes. **p < 0.01 vs. the Normal groups, ^##^ *p* < 0.01 vs. the KOA group. (E) Relative gene expression of MMP3, MMP13, ADAMTS4 and ADAMTS5 in chondrocytes. **p < 0.01 vs. the Normal groups, ^##^ *p* < 0.01 vs. the KOA group. (F) Content of MMP3, MMP13, ADAMTS4 and ADAMTS5 in cell supernatant. **p < 0.01 vs. the Normal groups, ^##^ *p* < 0.01 vs. the KOA group.

In vitro, TNN siRNA was used to silence TNN expression in chondrocytes. Immunofluorescence of chondrocytes (Figure 3A,B) showed the relative fluorescence intensity of TNN was increased in the KOA group compared with the Normal (*p* < 0.01) and decreased in the KOA + TNN siRNA group compared with the KOA (*p* < 0.01), not only confirming the successful establishment of the KOA model in vitro but also verifying the transfection efficiency of siRNA. In consistent with the results of in vivo, both gene and protein levels (Figure 3C–E) of MMP3, MMP13, ADAMTS4 and ADAMTS5 were up‐regulated in the KOA group compared with the Normal group (*p <* 0.01) and down‐regulated in the KOA + TNN siRNA group compared with the KOA (*p* < 0.01). Besides, the contents of the above proteolytic enzymes in the cell supernatant of each group (Figure 3F) also showed the same difference (*p* < 0.01).

### 3.4 RNA‐Seq of KOA Chondrocytes Revealed That TNN May Regulate AMPK‐PPARγ Signalling

**FIGURE 4:**
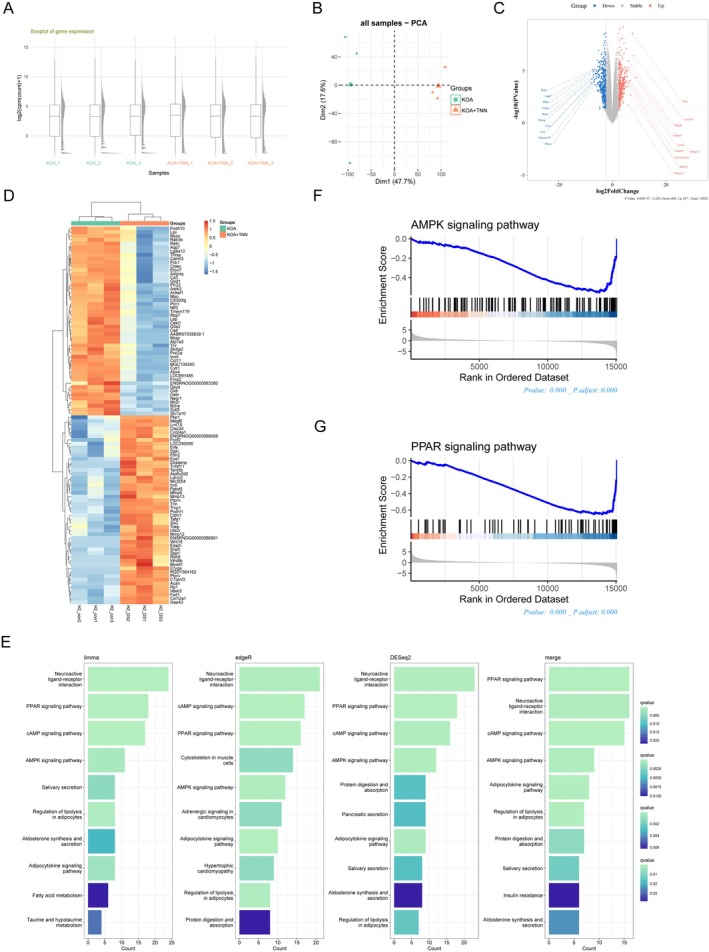
RNA‐seq of KOA chondrocytes revealed that TNN may regulate AMPK–PPARγ signalling. (A) The boxplot diagram reflected the sequencing depth of each tissue sample. (B) Individual‐PCA plot of gene expression in KOA (cyan) and KOA + TNN samples (orange) revealed strong clustering of samples by phenotype. (C) Volcano plot representation of DEGs analysis in KOA and KOA + TNN. (D) Heat map of down‐regulated DEGs, KOA vs. KOA + TNN. (E) KEGG analysis conducted by down‐regulated DEGs. (F) GSEA analysis of DEGs in ‘AMPK signalling pathway’. (G) GSEA analysis of DEGs in ‘AMPK signalling pathway’.

To further analyse the downstream target genes of TNN, we performed RNA‐sequencing on IL‐1β‐stimulated KOA chondrocytes (KOA group) and KOA chondrocytes treated with TNN (KOA + TNN group). The boxplot (Figure 4A) reflected that the sequencing depth of the samples is consistent, and PCA (Figure 4B) revealed strong clustering of samples by phenotype. Compared with the KOA group, the KOA + TNN group identified 437 up‐regulated genes and 498 down‐regulated genes; not unexpectedly, TNN was significantly up‐regulated in the KOA + TNN group, along with Mmp3, Mmp12, Mmp13, Tnfsf11 (Figure 4C). Notably, down‐regulated DEGs (Figure 4D), including Plin1, Lep, Lipi, were mainly enriched in ‘PPAR signalling pathway’, ‘AMPK signalling pathway’, ‘Adipocytokine signalling pathway’ in KEGG analysis (Figure 4E). GSEA plots evaluating the changes of related genes in the AMPK signalling pathway (Figure 4F) and PPAR signalling pathway (Figure 4G) showed that genes in these categories were down‐regulated in the KOA + TNN group. Since PPAR is widely considered to be one of the downstream molecules of AMPK, we speculated that TNN may regulate AMPK‐PPARγ signalling.

### 3.5 TNN Negatively Regulates AMPK‐PPARγ Signalling in KOA Cartilage Tissue

**FIGURE 5:**
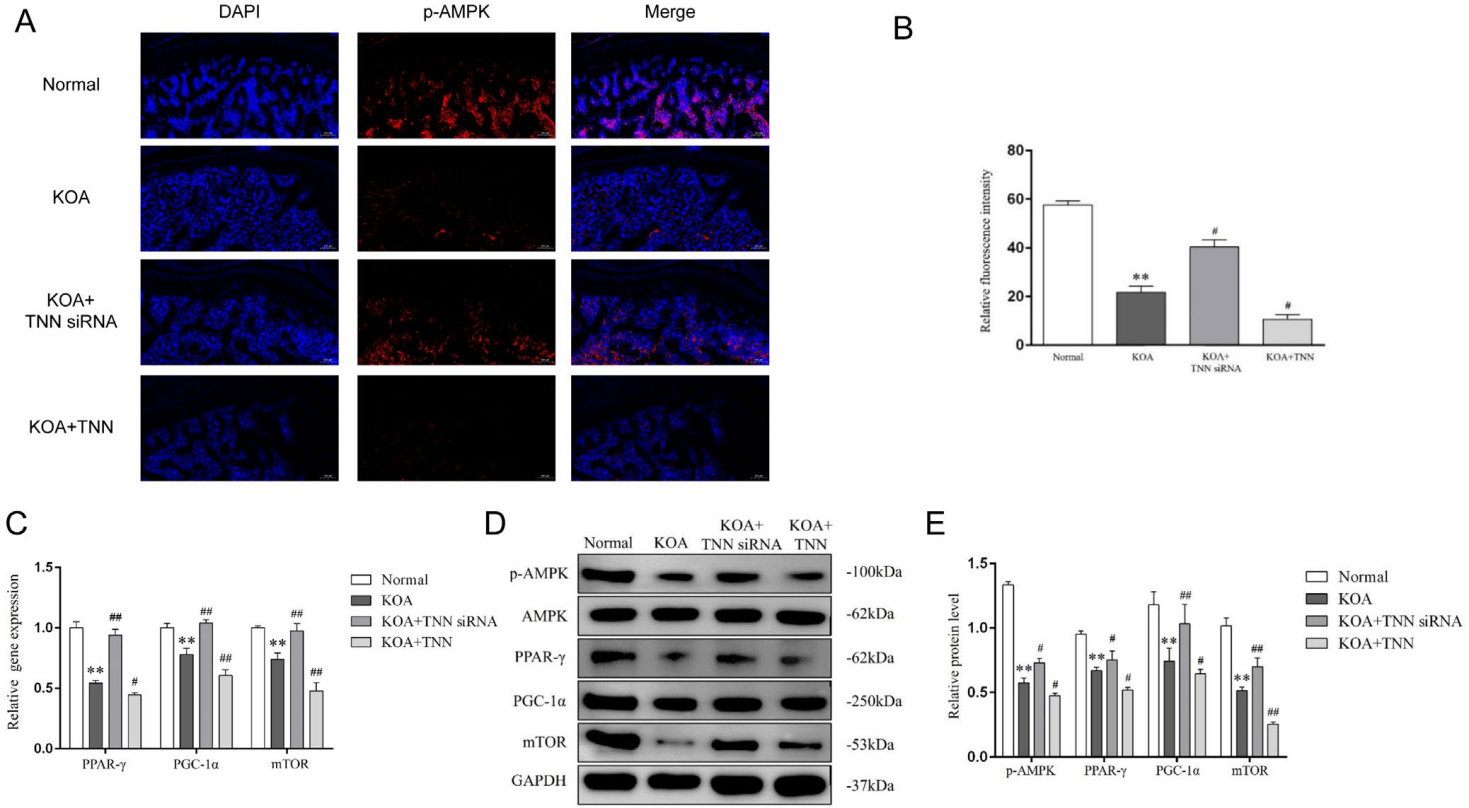
TNN negatively regulates AMPK‐PPARγ signalling in KOA cartilage tissue. (A) Representative p‐AMPK immunofluorescence of cartilage in each group, 100×, scale bar = 200 μm. (B) Relative fluorescence intensity of p‐AMPK. **p < 0.01 vs. the Normal groups, ^#^ *p* < 0.05 vs. the KOA. (C) Relative gene expression of PPAR‐γ, PGC‐1α and mTOR in cartilage. **p < 0.01 vs. the Normal groups, ^#^ *p* < 0.05, ^##^ *p* < 0.01 vs. the KOA group. (D) Typical protein bands of p‐AMPK, AMPK, PPAR‐γ, mTOR and PGC‐1α. (E) Relative protein level of p‐AMPK, AMPK, PPAR‐γ, PGC‐1α and mTOR in cartilage. **p < 0.01 vs. the Normal groups, ^#^ *p* < 0.05, ^##^ *p* < 0.01 vs. the KOA group.

To make one step further, p‐AMPK in cartilage tissues was marked with immunofluorescence (Figure 5A). In consistent with the RAN‐seq in chondrocytes, the relative fluorescence intensity of p‐AMPK (Figure 5B) was decreased in the KOA group compared with the KOA + TNN (*p* < 0.05), and compared with the KOA group, intervention with lentivirus mediated TNN siRNA significantly increased p‐AMPK fluorescence intensity (*p* < 0.05). Besides, the inhibition of TNN reversed the down‐regulated gene expression of PPAR‐γ, PGC‐1α and mTOR (Figure 5C) in KOA cartilage tissue (*p* < 0.01), while the TNN recombinant protein further intensified their down‐regulation (*p* < 0.05). The protein expression (Figure 5D,E) of the above substances showed the same trend as gene expression among the groups, as well as p‐AMPK (*p* < 0.05).

### 3.6 TNN Negatively Regulates AMPK‐PPARγ Signalling in KOA Chondrocytes

**FIGURE 6:**
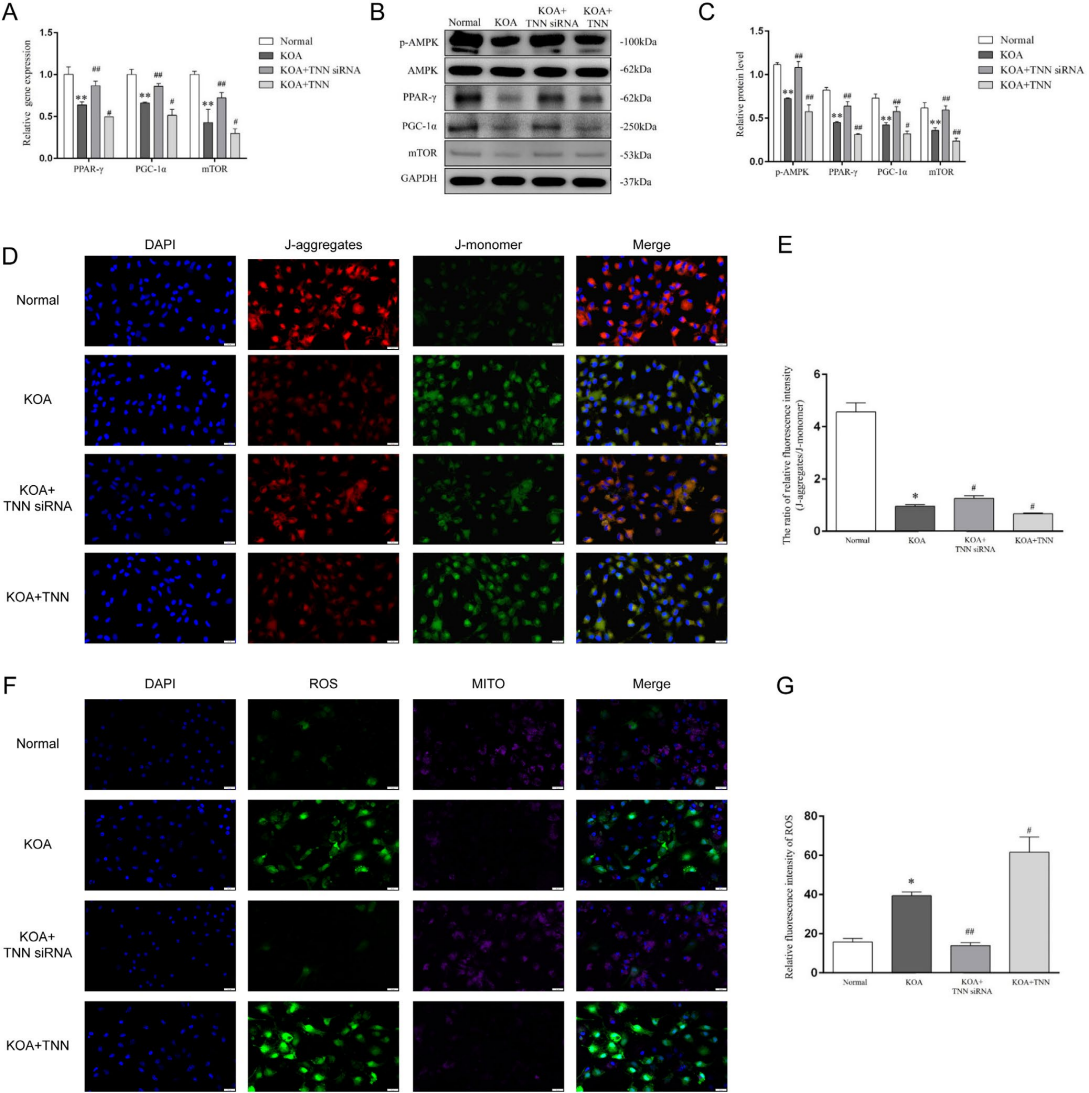
TNN negatively regulates AMPK‐PPARγ signalling in KOA chondrocytes. (A) Relative gene expression of PPAR‐γ, PGC‐1α and mTOR in chondrocytes. **p < 0.01 vs. the Normal groups, ^#^ *p* < 0.05, ^##^ *p* < 0.01 vs. the KOA group. (B) Typical protein bands of p‐AMPK, AMPK, PPAR‐γ, mTOR and PGC‐1α. (C) Relative protein level of p‐AMPK, AMPK, PPAR‐γ, PGC‐1α and mTOR in chondrocytes. **p < 0.01 vs. the Normal groups, ^#^ *p* < 0.05, ^##^ *p* < 0.01 vs. the KOA group. (D) Representative immunofluorescence of JC‐1 probes in each group, 400×, scale bar = 20 μm. (E) J‐aggregates/J‐monomer ratio of relative fluorescence intensity. *p < 0.05 vs. the Normal groups, ^#^ *p* < 0.05 vs. the KOA. (F) Representative immunofluorescence of mitochondrial ROS in each group, 400×, scale bar = 20 μm. (G) Relative fluorescence intensity of mitochondrial ROS. *p < 0.05 vs. the Normal groups, ^#^ *p* < 0.05, ^##^ *p* < 0.01 vs. the KOA.

Finally, we investigated the regulation of TNN on AMPK‐PPARγ signalling in vitro. Consistent with the results of in vivo, TNN siRNA reversed the down‐regulated expression of p‐AMPK, PPAR‐γ, PGC‐1α and mTOR (Figure 6A–C) in KOA chondrocytes (*p* < 0.01), while the TNN recombinant protein further brought them down (*p* < 0.05). Considering that AMPK‐PPARγ signalling affects mitochondrial function, we further evaluated mitochondrial function using JC1 probes (Figure 6D) and observed the release of mitochondrial reactive oxygen species (ROS, Figure 6F). The J‐aggregates/J‐monomer ratio of relative fluorescence intensity (Figure 6E) was decreased in the KOA group compared with the Normal (*p* < 0.05), suggesting the impaired mitochondrial function in KOA chondrocytes. TNN siRNA ameliorated impaired mitochondrial function, while TNN recombinant proteins further aggravated this impairment (*p* < 0.05). Not unexpectedly, the differences in mitochondrial ROS (Figure 6G) among the groups showed the opposite trend (*p* < 0.05).

## 4 Discussion

In this study, we attempted to get a better understanding of which genes are involved in cartilage damage in KOA. RNA seq for cartilage tissues obtained from the Normal group and KOA group revealed TNN was significantly up‐regulated in the KOA group, and the GO enrichment of DEGs in ‘extracellular matrix’, ‘extracellular matrix organization’, ‘collagen‐containing extracellular matrix’, ‘extracellular matrix structural constituent’ also proved the reliability of the results. Subsequently, in vivo and in vitro experiments were performed and demonstrated that TNN inhibition could reduce the expression of MMPs and ADAMTSs in cartilage and delay the progression of cartilage damage in KOA. Furthermore, RNA‐seq on IL‐1β‐stimulated chondrocytes and chondrocytes treated with TNN recombinant protein after IL‐1β stimulation were conducted to analyse the downstream target genes of TNN; KEGG analysis confirmed that AMPK‐PPARγ signalling might be the downstream pathway of TNN and might be negatively regulated by TNN. Under this circumstance, we investigated the regulation of TNN on AMPK‐PPARγ signalling in vivo and in vitro. Considering that AMPK‐PPARγ signalling affects mitochondrial function, mitochondrial ΔΨm and ROS were also detected in vitro.

Of the four tenascins, TNN is the least understood. It was first discovered in the zebrafish and later in mouse, expressed primarily in developing and mature bone, in a subset of stem cell niches, and in the stroma of many solid tumours [25, 26]. Like tenascin‐C, the fibrinogen‐related domain of TNN can bind and activate TLR4, which indicates TNN may play a role in inflammation and inflammatory diseases [27]. In addition, its prominent expression in the periosteum, a dense connective tissue around the bones containing progenitor cells that develop into osteoblasts, and at sites of osteogenesis, suggests a role for TNN in bone repair and remodelling [28]. This hypothesis seems to be confirmed by Kimura H et al. In their study, cells in the newly formed perichondrium surrounding the cartilaginous callus expressed TNN during bone fracture repair; besides, TNN inhibited osteoblastic proliferation and suppressed canonical Wnt signalling, which stimulates osteoblastic differentiation [29]. As is well‐known, KOA is closely related to inflammation and bone metabolic remodelling; the inflammatory microenvironment is considered to be the initiator and promoter of the progression of cartilage and subchondral bone pathology in KOA [30]. Based on previous studies of TNN in the field of inflammation and bone remodelling, the involvement of TNN in the pathological progression of KOA is no longer difficult to understand. In this study, TNN was proved to be a novel marker of cartilage damage upregulated in KOA according to RNA‐seq, and TNN inhibition could reduce the expression of MMPs and ADAMTSs in cartilage and delay the progression of cartilage damage in KOA.

Further on, we identified AMPK‐PPARγ signalling as a potential downstream pathway for TNN to accelerate cartilage damage with RNA‐seq. AMPK is a heterotrimeric complex that senses low cellular ATP levels and responds to the decreasing ATP to AMP ratio by regulating metabolic enzymes to boost ATP generation and suppress ATP consumption, which is an essential regulator for energy homeostasis [31]. Decreased phosphorylation of AMPK reduces PPAR‐γ DNA binding activity and protein expression, subsequently reduces transcriptional inhibition of NF‐κB, and increases the release of inflammatory mediators [32]. Additionally, dysfunction of AMPK is correlated with impaired autophagy, abnormal endoplasmic reticulum stress, and increased oxidative stress, which are often achieved by AMPK regulation of PGC‐1α and mTOR [2, 32]. Compared with normal joint tissues, the articular cartilage of KOA patients and surgically‐induced KOA mice have significantly reduced AMPK phosphorylation [33, 34]. Further studies showed that increased AMPK phosphorylation promotes the expression of the autophagy‐related protein LC3II through mTOR in chondrocytes, activates lysosome activity, promotes the formation of autophagy‐associated lysosomes, and vitamin D may protect KOA chondrocytes from apoptosis by intervening in the above links [35]. In another study, metformin does play a chondroprotective effect through activation of AMPK signalling, while also significantly increasing the expression of p‐AMPK and total AMPK protein levels in dorsal root ganglion tissues; in turn, the pain behaviour of the KOA mice was relieved [36]. Besides, it has been proven that PPAR‐γ activation inhibited chondrocytes pyroptosis to alleviate KOA through PGC‐1α/ΔΨm pathways, and the role of PPAR‐γ activation in blocking the nuclear factor erythroid‐2‐related factor/NLRP3 pathway has also been demonstrated in the same study [37]. Consistent with previous studies, we found that recombinant TNN negatively regulates AMPK/PPARγ signalling in vivo and ex vivo in this study, whereas TNN siRNA increases AMPK phosphorylation, activates downstream PPARγ signalling, and modulates mitochondrial membrane potential and ROS production in chondrocytes, which may participate in KOA cartilage injury.

Nevertheless, our study has certain limitations. First, we did not perform protein–protein interaction assays and therefore lack direct evidence that TNN acts on AMPK. It remains to be determined whether TNN may potentially affect AMPK phosphorylation in a non‐specific manner, or whether the AMPK–PPARγ pathway is indeed the primary downstream mechanism through which TNN exerts its deleterious effects. Secondly, as TNN is endogenously elevated in KOA cartilage, we currently lack clinical data to determine whether TNN levels are associated with the severity of cartilage damage. Finally, the effects of TNN on mitochondrial membrane potential and ROS levels in chondrocytes only partially reflect mitochondrial function; more robust data on mitochondrial biogenesis and energy metabolism remain to be elucidated. Further experiments are needed to address these questions.

In summary, we found evidence of differential expression of TNN in articular cartilage of KOA rats and proved upregulated TNN accelerated cartilage damage via negative regulation of AMPK‐PPARγ signalling. Our study provides new insights into the pathological mechanisms of KOA cartilage damage, as well as new directions for pharmacological intervention.

## 5 Conclusions

In conclusion, our study innovatively unveils increased TNN in KOA that accelerates cartilage damage, and this damage‐promoting effect is achieved by negative regulation of AMPK‐PPARγ signalling.

## Author Contributions

**Zhiyu Chen:** data curation, writing‐original draft. **Gean Wu:** validation, writing – original draft. **Yizhe Fan:** investigation, resources. **Chao Li:** investigation. **ChenHao Wang:** investigation. **Chengyi Yang:** formal analysis. **Shixiang Wu:** formal analysis. **Zhaoshun Wu:** resources. **Peng Wang:** visualisation. **Yafeng Zhang:** conceptualization, methodology, supervision. **Wulin You:** project administration, data curation, writing – review and editing, funding acquisition.

## Funding

The current work was supported by the Top Talent Support Program for young and middle‐aged people of Wuxi Health Committee (BJ2023070); Jiangsu CM Clinical Innovation Center of Degenerative Bone & Joint Disease (20210405); Jiangsu Provincial Traditional Chinese Medicine Science and Technology Development Program Project (MS2024059); Wuxi Municipal Special Project for the Development of Traditional Chinese Medicine Science and Technology (ZYQN202402); Wuxi Science and Technology Innovation and Entrepreneurship Funds for the Ninth Batch of Science and Technology Development Plan Projects (K20241019).

## Ethics Statement

This study was established and authorised by the Ethics Committee of Wuxi Affiliated Hospital of Nanjing University of Chinese Medicine (Approval number: 2024(dongwu)‐030‐01).

## Consent

The authors have nothing to report.

## Conflicts of Interest

The authors declare no conflicts of interest.

## Data Availability

The data that support the findings of this study are openly available in Science Data Bank www.scidb.cn at https://doi.org/10.57760/sciencedb.24274.
